# Supplementary material for: Overdue Calcium Oscillation Causes Polyspermy but Possibly Permits Normal Development in Mouse Eggs
Source: Int J Mol Sci. 2023 Dec 24;25(1):285. doi: 10.3390/ijms25010285 (PMC10779150; doi:10.3390/ijms25010285)
Supplement: Supplementary file 1 [file ijms-25-00285-s001.zip › ijms-2719226-supplementary.pdf]

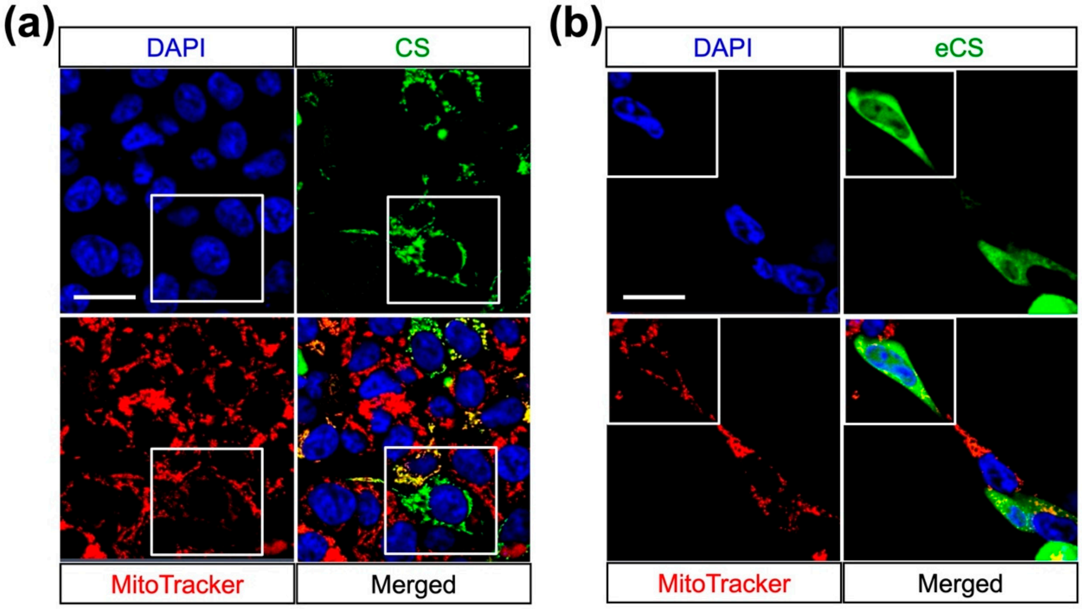

Figure S1. Localization of CS and eCS fused with EGFP. Red CMXRos. Green: CS and eCS fused with EGFP; red: mitochondria; blue: DAPI. Boxes were enlarged in Figure 1b. Scale bars: 10 μm.

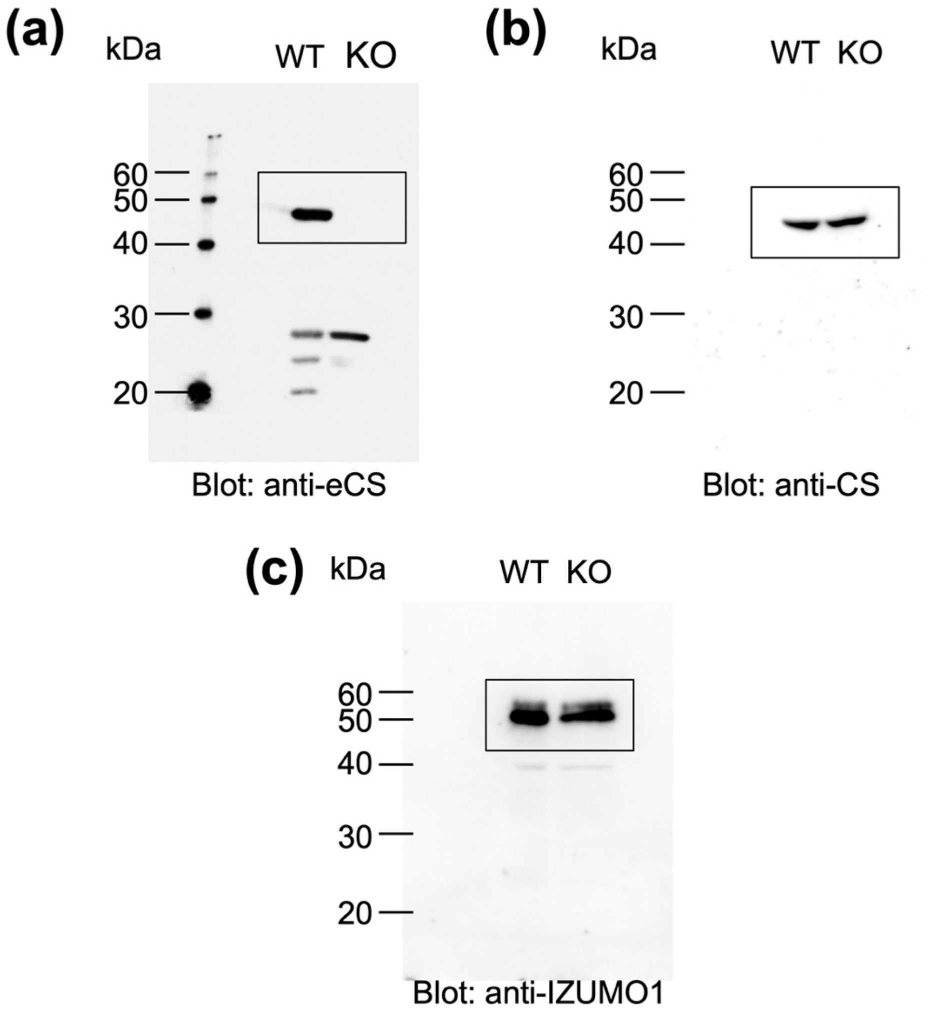

Figure S2. Immunoblotting of sperm with anti-IZUMO1, CS and eCS Abs. Boxes were shown in Figure 4d.

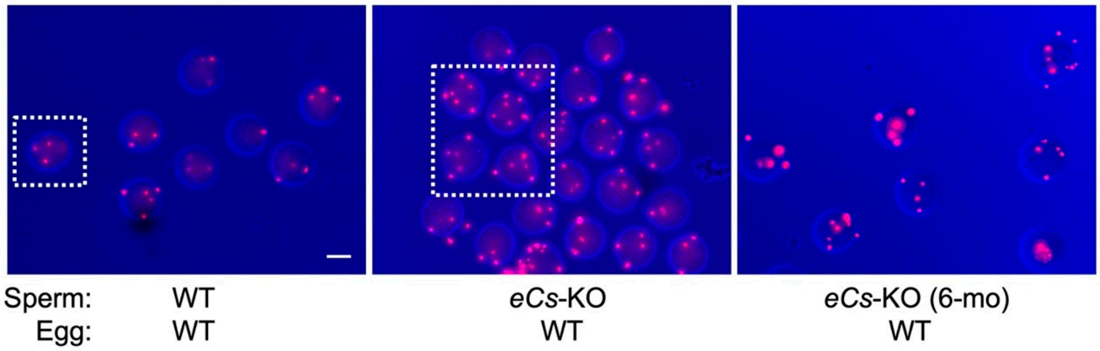

Figure S3. Polyspermic eggs. Boxes were enlarged in Figure 5b. Scale bar: 50 µm.
